# Evolving California genotypes of *Avena barbata* are derived from multiple introductions but still maintain substantial population structure

**DOI:** 10.7717/peerj.633

**Published:** 2014-11-04

**Authors:** Kate Crosby, Taylor O. Stokes, Robert G. Latta

**Affiliations:** Department of Biology, Dalhousie University, Halifax, NS, Canada

**Keywords:** *Avena barbata*, Native range, Multiple introductions, Chloroplast DNA, Latitudinal cline, Phylogeography, Invasive species, Genome size

## Abstract

Multiple introductions are thought to enhance the chance of successful colonization, in part because recombination may generate adaptive variation to a new environment. *Avena barbata* (slender wild oat) is a successful colonist in California, historically noted for striking genetic divergence into two multilocus genotypes, but is still undergoing adaptive change. We sought to understand whether multiple introductions might be contributing to this change. We used cpDNA phylogeography of *A. barbata* within its home range and in its invaded range in California to determine the minimum number of separate introductions, and the spatial distribution of these introduced lineages. We collected from sites throughout the state of California, where it is an invasive species. Accessions from a representative portion of *A. barbata*’s full native range were obtained from germplasm repositories. We sequenced seven intergenic chloroplast DNA loci for *A. barbata* individuals both in California (novel geographic range) and its ancestral range. 204 individuals were assayed for chloroplast haplotype within California using single strand conformational polymorphism SSCPs. Genome size was determined by flow cytometry. Californian accessions are tetraploid as expected, but their genome sizes were smaller than the Old World accessions. There were three haplotypes present in California that were identical to haplotypes in the native range. Within California, the presence of multiple haplotypes at a site was observed primarily in Northern and Central populations. Between populations there was still substantial structure with *F*_ST_ ∼ 0.33, due to a shallow latitudinal cline caused by a preponderance of xeric haplotypes in Southern California. There was a minimum of three seed introductions to California. Recombination is thus likely to occur, and contribute to adaptation in new range in this highly-selfing, invader.

## Introduction

Introduced and invasive species are likely to have to adapt to novel conditions (Dlugosch & Parker, 2008; Prentis et al., 2008), and the first requirement of adaptation is access to a pool of genetic variation. The pool of genetic variation can be increased through introductions from multiple sources. Typically, adaptive genetic variation is much reduced in the new range compared to the home range, as many invasive species reproduce primarily via selfing or are facultatively asexual during colonization of the new range (Baker, 1955; Baker, 1967; Price & Jain, 1981; Barrett & Colautti, 2008). During invasions, the main advantage of selfing is that individuals need not depend on pollinators or other conspecifics to pollinate with for persistence or spread. Indeed, in selfing or clonal species, one or a few introductions could be enough to allow for successful establishment and persistence—even on a global scale (Le Roux et al., 2007). By contrast, outcrossing species generate new multilocus genotypes with each round of outcrossing, provided that enough individuals of different genotypes have been introduced that mates are available and to avoid inbreeding depression.

Self-fertilizing species typically suffer less from the effects of inbreeding depression than outcrossers (Husband & Schemske, 1996), but they may still require adaptive genetic variation in order to respond to novel environmental conditions. It has been suggested that multiple introductions of different genetic variants allow selfing species the opportunity for occasional outcrossing and recombination in new environments (Ellstrand & Schierenbeck, 2000; Schierenbeck & Ellstrand, 2009). Assessing the minimum number of introductions in a new environment is thus critical to evaluating how important the amount of genetic variation may be for self-fertilizing species in the new range, which will ultimately determine how much effective recombination is possible in the new range.

Californian populations of the highly-selfing (Marshall & Allard, 1970), tetraploid (Hutchinson et al., 1983), invasive annual grass, *Avena barbata* Pott ex Link are thought to have been introduced from the Iberian Peninsula roughly two centuries ago during Spanish colonization (Jain & Marshall, 1967; Garcia et al., 1989). *Avena barbata* became widely known for a number of pioneering observations in the 1970s (Clegg & Allard, 1972; Allard et al., 1972; Hamrick & Holden, 1979) which found that there were predominantly two multilocus genotypes of *A. barbata* in California. Each genotype was monomorphic for a set of five allozyme loci, and few recombinants were found. One genotype was found in moist environments, while the other occurred in more arid environments, a pattern repeated at both large (Clegg & Allard, 1972; Allard et al., 1972) and small geographic scale (Hamrick & Holden, 1979), leading to the interpretation that these represented locally adapted ‘mesic’ and ‘xeric’ ecotypes. The allozyme combinations characteristic of the original Californian genotypes were not found in native Iberian populations (Garcia et al., 1989). This suggests that recombination among separate genotypes was part of the evolutionary history of *A. barbata* in California.

Past experimental work in which a novel environment was imposed in the greenhouse demonstrated that recombination between the two genotypes produced a few hybrid recombinants that were more fit than the parents (Johansen-Morris & Latta, 2006; Johansen-Morris & Latta, 2008). Additionally, a previous four-year field common garden, reciprocal transplant experiment of parental genotypes to wet and dry environments found that the mesic genotype, and a few recombinants are consistently more fit than the xeric (Latta, 2009). Thus, the mesic could be displacing the xeric genotype, or a recombinant could be displacing both as the prominent genotype throughout California. Collectively, this evidence does not support the idea that the two genotypes are locally adapted to moist and arid environments as Allard’s work suggested (though we retain the names ‘mesic’ and ‘xeric’ here), but rather that adaptive change is still occurring in California. This continuing evolution could be due to recombination between several different genetic variants as demonstrated by Johansen-Morris & Latta (2006), Johansen-Morris & Latta (2008) and/or the superior fitness of one lineage (likely the mesic) (Latta, 2009).

The first step in assessing the role of intermixing and recombination is to determine the minimum number of introductions to California and their spatial distribution. We constructed a cpDNA phylogeny of accessions from across *A. barbata*’s native range as well as its invaded range in California to determine the minimum number of introductions to California. In most plants, organelle genomes of both the chloroplast and mitochondrion are maternally inherited (Corriveau & Coleman, 1988; Zhang, Liu & Sodmergen, 2003), and provide a useful trace of seed introduction during colonization. We hypothesize that the mesic and xeric genotypes are the result of at least two separate seed (maternal) introductions to California from the native range. We sought to characterize the geographic distribution and the degree of spatial overlap of these introductions within California. As *A. barbata* is highly selfing, pollen movement is limited and recombination is restricted until a new genotype arrives in the population via seed (maternal) gene flow. Limited seed migration would tend to restrict levels of spatial intermixing, and we expected that for a selfing species most genetic diversity would occur amongst rather than within populations (Hamrick & Godt, 1996). We assayed a wider panel of individuals across California sampled from both within and amongst populations to assess the validity of this expectation. Future studies using nuclear loci will examine the degree of recombination among genotypes in California. In order to rule out novel polyploid formation in an invader (often a result of hybridization (Novak, Soltis & Soltis, 1991; Daehler & Strong, 1997)), we estimated the ploidy of a subset of accessions from both ranges using flow cytometry. As a cpDNA phylogeny of most of the species in the genus *Avena* is available (based on the trnL-F/trnF-R intergenic region—Peng et al., 2010), we placed a root to our own cpDNA tree with a direct comparison of chloroplast sequence data and ploidy estimates.

## Methods

### Source of material

Old World accessions of *A. barbata* were donated by Agriculture Canada’s Plant Genome Resources of Canada (PGRC) germplasm station in Saskatoon, Saskatchewan, Canada, and the USDA National Small Grains Collection (NSGC) in Aberdeen, Idaho, USA. We attempted to obtain accessions from across *A. barbata’s* range in the Old World, but with particular emphasis on the Iberian Peninsula (Spain and Portugal) as this is thought to be the origin of Californian *A. barbata* (Jain & Marshall, 1967; Garcia et al., 1989; Minnich, 2008). Official repository accession names, our own sample code names, GenBank accession numbers along with sampling locations and other information are given in Supplemental Information 1A.

In May 2010, we carried out an extensive geographic survey of *A. barbata* sites in California, USA. We collected seeds from 95 sites matching locations described in three dissertations (Clegg, 1972; Miller, 1977; Hutchinson, 1982) that studied allozyme variation in the 1970s. These sites represent the full range of growing conditions for *A. barbata*, and will be used for future studies comparing present day to past genotypic composition. We walked ∼3–5 m between each individual at a collection site to avoid sampling close relatives. In addition, we included in the analysis the mesic and xeric accessions used by Gardner & Latta (2006) to create a genetic mapping population of *A. barbata*—these seeds were kindly provided by P. Garcia from collections made in California during the 1980s, and had previously been genotyped using the original set of five allozyme loci (Latta et al., 2004). Seeds were germinated following our lab’s standard protocol (Latta et al., 2004), and then planted in the greenhouse at Dalhousie University. Californian populations were grown in June 2010 and June 2011, while seeds from germplasm repositories were grown in June 2011 and June 2012.

### Ploidy assessment

Forty-eight accessions from the Old World, and 24 Californian individuals were assayed for ploidy via flow cytometry. Flow cytometry was performed with a BD FACSCalibur flow cytometer at the University of Guelph, Ontario, Canada with CellQuest Pro software (BD Biosciences, San José, USA). All assays came from fresh leaf tissue harvested from young plants (10–20 cms in height) from the greenhouse at Dalhousie University and were shipped to Guelph in moist paper towels. Sample preparation was modified slightly from a previous protocol (Doležel, Greilhuber & Suda, 2007). The DNA content standard used was *Vicia faba* (26.90 pg/2C) (Doležel, Cíhalíková & Lucretti, 1992). Approximately 0.5 cm^2^ of *V. faba* and 1.2 cm^2^ of *A. barbata* were chopped with a razor blade, and was placed in cold extraction buffer for staining (100 µg/ml propidium iodide and 50 µg/ml RNAse—in this study LB01 buffer was used (Doležel, Greilhuber & Suda, 2007)). The FL-2 peak analysis program was used to infer ploidy from 2C DNA content measurements of each individual against the DNA content standard. To examine whether there was any difference in DNA content (pg/2C) between California and the Old World, we performed a one-way two-sample randomization test based on 1000 Monte-Carlo re-samplings of the approximate distribution using the R-package ‘coin’ (Zeileis et al., 2008).

### Chloroplast DNA variation

To create the phylogeny, we obtained cpDNA sequences from 49 Old World and 32 Californian accessions, which included two Mesic and two Xeric standards (the parents of the mapping population described in Latta et al. (2004)). We chose California sites that formed North-South and East-West transects by choosing at least one accession from sites previously sampled by Clegg (1972) and Hutchinson (1982). DNA extraction was carried out from leaf tissue following a slightly modified protocol of plant DNA extraction (Dellaporta, Wood & Hicks, 1983); we also employed an expedient protocol optimized for seeds (Ivanova, Fazekas & Hebert, 2008) for 10 samples.

We used seven previously described primer pairs (Ebert & Peakall, 2009; Taberlet et al., 1991) for the large single copy (LSC) region of the chloroplast (Table 1). All PCR products were visualized on a 1.5% agarose TAE gel run at 60 mA, 100 V for approximately 1.5 h. Sanger cycle-sequencing reactions for PCR products with single, clear bands were carried out by MacrogenUSA Inc. For each primer pair, we used only the forward primer for sequencing, with the exception of the *trnT-F/trnL-R* fragment, which was bi-directionally sequenced. To infer a root for our final phylogeny we screened one individual from each cpDNA haplotype using *trnL-F/trnF-R* (Taberlet et al., 1991), and compared this to Peng et al.’s (2010) phylogeny of the genus *Avena*.

**Table 1:** Summary of intergenic cpDNA loci used. Intergenic locus region target, the primer pair (as given in reference), the number of variable characters for each locus, and the length of the fragment uploaded to GenBank. Sequencing primers are underlined. The eighth and last intergenic locus (trnL (UAA) 3^′^exon) is italicized because we only screened unique haplotypes among accessions from our dataset to ascertain the root of our tree see Supplemental Information 1C.

Intergenic regions targeted	Primer pair	Number of variable characters	Length of fragment in *A. barbata* (bp)	Reference
trnQ (UUG)–psbK	ANU11-L/ANU-12R	5	408	Ebert & Peakall (2009)
atpB–rbcL^a^	ANU67-L/ANU68-R,	7	901	Ebert & Peakall (2009)
psaI - ycf4^a^	ANU73-L/ANU74-R	3	356	Ebert & Peakall (2009)
psaJ–rpl33	ANU83-L/ANU84-R	1	221	Ebert & Peakall (2009)
rpl33–rps18	ANU85-L/ANU86-R	4	595	Ebert & Peakall (2009)
trnT (UGU)	trnT-F/trnL-R	4	510	Taberlet et al. (1991)
trnL (UAA) 5′exon	trnT-F/trnL-R	4	259	Taberlet et al. (1991)
*trnL (UAA)* 3′*exon*	*trnL-F**/trnF-R*	*3*	*730*	Taberlet et al. (1991)

**Notes.**

a These loci were used in SSCP analysis.

To assess the distribution of haplotypes in the introduced range, we expanded our screening to more accessions within California, using single strand conformational polymorphism (SSCP). SSCPs are an efficient, and cost-effective method for screening many samples that isolate single nucleotide polymorphisms (SNPs) (Gasser et al., 2007). SSCPs were used for two chloroplast intergenic regions that displayed variation within California. Two Californian haplotypes were separated by an SNP at the intergenic region spanning *atpB* and *rbcL*. A third Californian haplotype differed from the first two at several loci of which we screened an indel for the intergenic region between *psaI* and *ycf4*. We performed double restriction digest of locus *atpB-rbcL* amplicons, with *HpaII* and *RsaI*, and of locus *psaI-ycf4* with *SspI* and *AluI*. These enzymes were chosen from the chloroplast sequence data to isolate the SNP and indel into smaller fragments conducive to SSCP assays. The fragments from double digests were denatured for 10 min at 95 °C, snap frozen, and run on non-denaturing polyacrylamide gels for 17.5 h at 502 V, 14 mA, and a constant wattage of 8 W. We attempted to screen 5–10 accessions from sites that had at least one individual sequenced for chloroplast loci, and one accession at each of the remaining 71 sites. This two-level sampling scheme allowed us to evaluate the potential of admixture within sites, and also broad population structure between sites in California. In total, 204 samples were screened for SSCPs. All of the Californian accessions for which we had already obtained chloroplast sequence data were also assayed with SSCPs, and we confirmed the accuracy of SSCP to relevant loci (Tables S1 and S2).

### Analysis

Trace files for each chloroplast locus were imported to Geneious v. 5.4.4, and aligned with each other using the MUSCLE algorithm within Geneious default settings, and confirmed by eye. Traces with QV scores <20 were discarded and not used in further analyses. Every character change (SNP or single indel) for each chloroplast locus was treated as an independent binary character. The only exception was the trnL-F (UAA) locus, which had several indels of multiple adjacent basepairs; these were treated as one multistate character. All polymorphic sites were then used to construct a maximum-likelihood phylogenetic tree using PhyML 3.0 (Guindon et al., 2010), iterated for 10,000 bootstraps. In order to doubly verify cpDNA tree topology, we also constructed a Bayesian phylogenetic tree using Mr. Bayes v. 3.2. Chloroplast trace files were blasted and annotated using the web server tool CpGAVAS (Liu et al., 2012) and uploaded to NCBI using the web server tool—BankIt.

For the Californian accessions assayed with SSCPs, we modeled the relative frequency of chloroplast haplotype at a site with respect to latitude using a generalized linear binomial model using the R package ‘lme4’ (Bates, Maechler & Dai, 2008). For this approach, any samples in the same population were treated as non-independent observations. Within California we estimated the overall *F*_ST_ for individuals at geographic sites assayed with SSCPs (*n* = 204).

## Results

### Ploidy estimations

**Figure 1:**
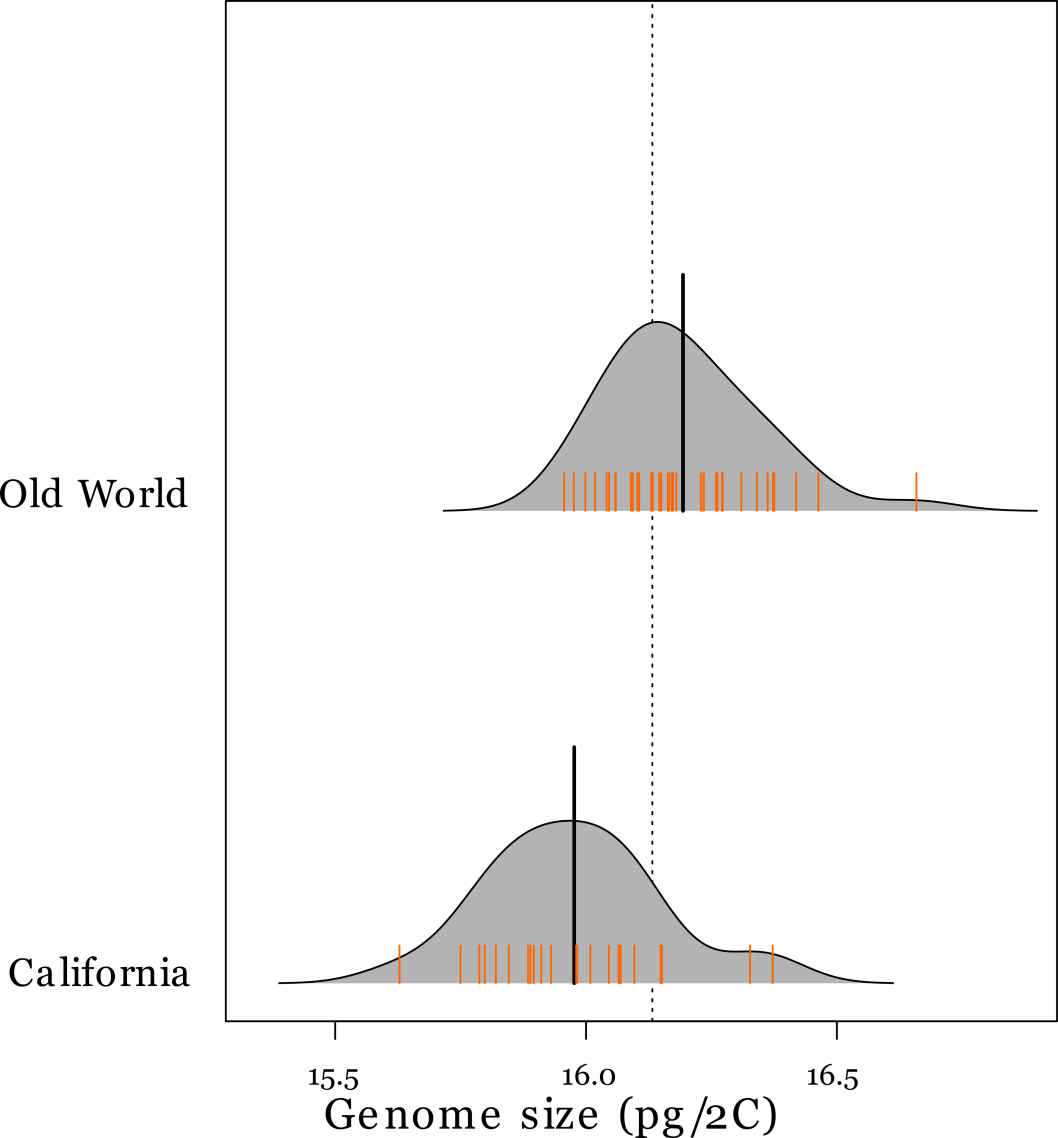
Genome sizes of tetraploid *Avena* species in the Old World and California. Probability density plot (grey-shaded region) and rug plot (orange hatch marks) of genome size values (pg/2C) for tetraploid *A. barbata* from the Old World (*n* = 40), and California (*n* = 24). The thick black line in the middle of each density plot is the median value for genome size. The dotted line is the overall median value.

**Figure 2:**
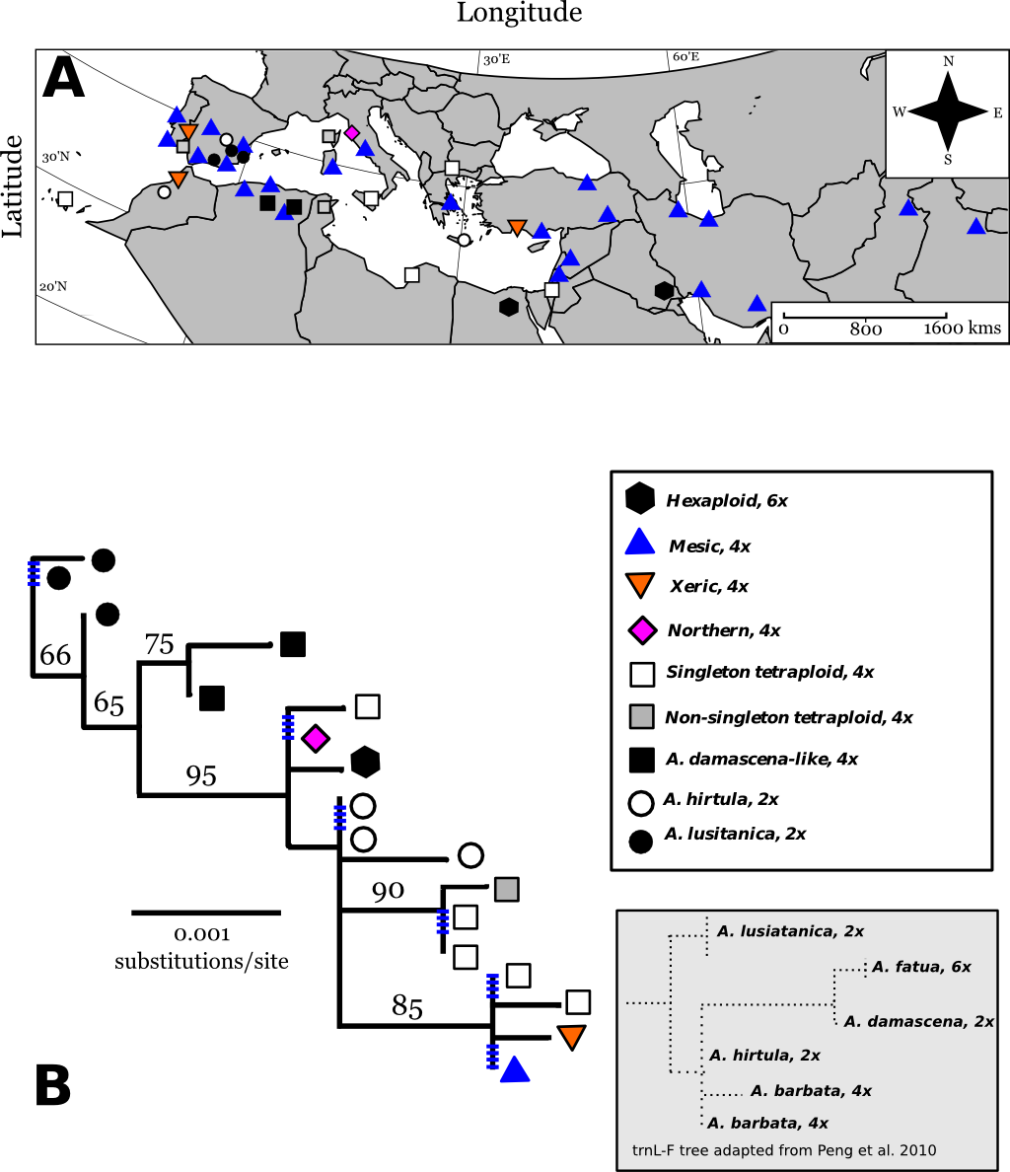
Map of Old World Avena accessions and cpDNA phylogeny juxtaposed with previous cpDNA phylogeny by Peng et al. (2010). Map (Lambert azimuthal equal-area projection) of the Old World accessions (A). The haplotypes in colour on the map represent the accessions that occur in both the Old World and California. Maximum-likelihood phylogenetic tree based on 100,000 re-samplings (B). Bootstrap support is indicated at nodes. The tree was constructed using all informative chloroplast sites of seven loci. The ploidy for each haplotype is mapped onto the tree, not included as a character in the phylogeny. Inset tree is a drawn rough approximation of Peng et al. (2010) tree for context in explaining our phylogeny’s hypothesized rooting. Blue hatched marks are collapsed branches, generally within a clade. Map of broad categories chloroplast haplotypes of Old World (European and Asian) wild oat accessions. “Singleton tetraploids” are not necessarily identical to each other, but all mesic and xeric haplotypes are identical to each other. See discussion for further elaboration on *A. damascena-like*, and *A. lusitanica* types.

Flow cytometry gave 2C DNA content clustered at values of 8, 16 and 24 pg (Table 2) implying variation in ploidy. *A. barbata* is tetraploid and 16 pg was the most common 2C content, so accessions that had approximately 8 pg and 24 pg were inferred to be diploids and hexaploids, respectively. These genome size estimates are well within previously reported estimates of other *Avena* species (Bennett & Leitch, 2005). All Californian accessions were tetraploid. However, we observed six diploid and two hexaploid accessions among the 48 Old World accessions. The ranges of the genome size (pg/2C) of Californian and tetraploid Old World accessions overlapped (Table 2), but Californian accessions have approximately 1.5% smaller genomes on average than tetraploids from the Old World (permutation test *Z* = 3.9626, *p* = 0.00001) (Fig. 1). Inferred ploidy is mapped onto our phylogeny (Fig. 2).

**Table 2:** Summary of genome size range from flow cytometry analyses. Broad grouping of accessions evaluated with flow cytometry, number of plants assayed, mean 2C DNA content (pg/2C), the minimum and maximum range of the mean DNA content, inferred ploidy, and standard error (SE). See Supplemental Information 1A–B for detailed genome size information.

Group	Sample size	Mean 2C DNA content (pg/2C)	SE	Range of 2C DNA content (pg/2C)	Inferred ploidy
All Californian accessions	24	15.97	0.03	15.62–16.36	4x
Tetraploid Old World accessions	40	16.21	0.03	15.68–16.69	4x
Diploid (Clade 1—Spain)	3	8.26	0.05	8.16–8.32	2x
Diploid (Clade 2—Morocco, Greece, Spain)	3	8.65	0.10	8.48–8.83	2x
Babylon, Iraq and Giza, Egypt	2	24.99	0.56	24.43–25.55	6x

### Phylogeography in the Old World

From seven chloroplast loci, we obtained 3250 bp of chloroplast sequence from which we found a total of 18 different cpDNA haplotypes across *A. barbata*’s range (Figs. 2A and 2B). Overall, there was very little cpDNA variation, and total chloroplast sequence divergence was 0.86% for all accessions in our phylogeny. Of the roughly six major clades in our phylogeny four were well supported (bootstrap support proportion of 75 or greater). However, the different haplotypes within each of these major clades were not well differentiated from each other with polytomies occurring in each clade (Fig. 2B). The most widely distributed haplotype in the Old World matched that of the mesic genotype. A haplotype matching that of the xeric genotype was very closely related to the mesic, being separated by only a single SNP. The xeric haplotype was present in three Mediterranean sites—for consistency, we refer to these haplotypes as “mesic” and “xeric”. Most other Old World haplotypes were unique single point occurrences (in Fig. 2A we label these as “singleton tetraploids”), with the exception of the identical haplotypes isolated from accessions in Portugal (CN25800), Corsica, France (PI337963), and Tunisia (CN19364).

Three diploid Spanish accessions belonged to one well-supported clade closely related to tetraploid Algerian accessions. The trnL-F/trnF-R sequences for these clades closely matched trnL-F/trnF-R sequences for the diploid *Avena lusitanica*, and the diploid *Avena damascena*, respectively (Peng et al., 2010). We therefore root our tree to these groups.

The other three diploids from Greece, Spain, and Morocco are basal to the clade containing the mesic and xeric haplotypes, along with other tetraploids (Figs. 2A and 2B). The trnL-F/trnF-R sequence of these diploids matches those of *A. hirtula* and *A. barbata*, which had identical trnL/trnF sequences in Peng et al. (2010) *A. hirtula* is the inferred diploid A genome ancestor of the AB tetraploid *A. barbata* (Allard et al., 1993). We suggest the diploid accessions in this clade are likely *A. hirtula*.

The two hexaploids (found in Iraq and Egypt) had the same haplotype, which is also basal to the main clade. These two hexaploids do not match the *trnL-F* sequences of hexaploid *A. fatua* (or any other *Avena* species) from Peng et al.’s (2010) phylogeny. Finally, all haplotypes in the main tetraploid clade containing the mesic and xeric haplotypes have *trnL-F* sequences matching those of Peng et al.’s (2010) *A. barbata* sequences.

### Introductions and population structure in California

**Figure 3:**
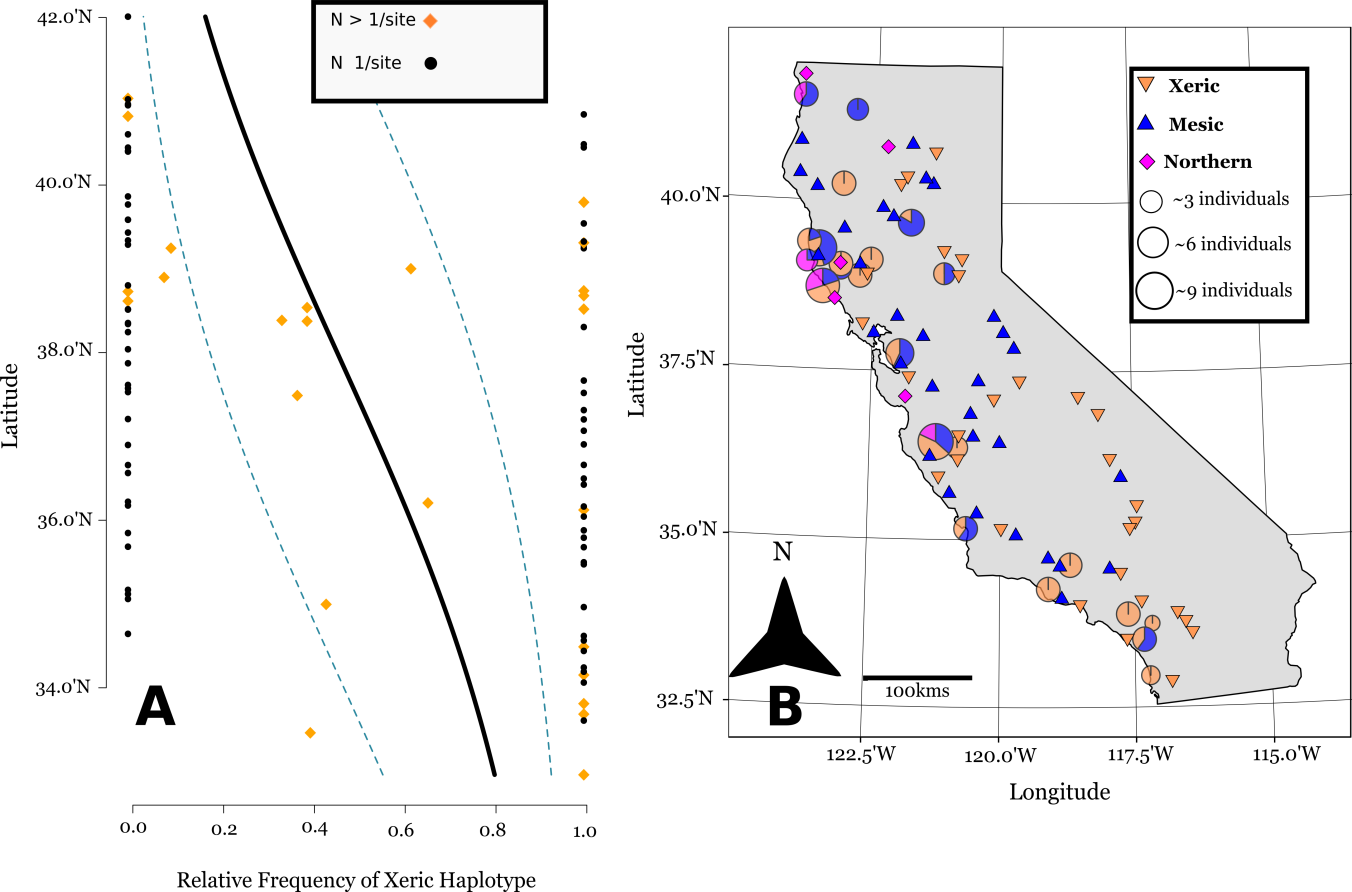
Linear logistic regression of latitudinal cline of cpDNA haplotypes in California. Linear logistic regression of the relative abundance of the xeric cpDNA haplotype on latitude, the 95% confidence bands based on the logistic distribution (A). Note the latitude is on the *y*-axis for comparison with the map. (B) shows the geographic distribution of haplotypes in California (Lambert azimuthal equal-area projection). Pie charts represent 24 sites where multiple individuals were assayed, and are sized relative to the number of individuals sampled at a site. Triangles represent 71 sites where one individual was sampled per site.

Three chloroplast haplotypes were identified in California. All three of these were also observed in the Old World (Figs. 2 and 3). The xeric and mesic haplotypes were of course present in California, given their association with the mesic and xeric allozyme genotypes of Allard et al. (1972). However, a third haplotype was found at sites in Northern California. One accession collected from Livorno, Italy had a chloroplast haplotype identical to that of this “Northern” haplotype (Fig. 2A), and this haplotype was distantly related to the mesic and xeric haplotypes.

The SSCP approach we used was able to differentiate between mesic and xeric haplotypes, and northern and mesic haplotypes (Supplemental Information). The SSCPs revealed 106 accessions possessing the xeric allele at marker ANU 67-L/68-R, distributed mostly at southern latitudes (Figs. 3A and 3B). We then were able to further differentiate 74 accessions that had the mesic allele from 24 Northern alleles at marker ANU 73-L/ANU 74-R in California (Fig. 3B). Chloroplast sequence divergence = 0.28% for Californian haplotypes, in comparison with 0.86% sequence divergence of all accessions, which is more than a 3x reduction in sequence divergence for Californian haplotypes.

Of the 24 sites that were screened for more than one individual, 12 of these sites were polymorphic (Fig. 3B). However, there is substantial spatial structure as to how this variation is distributed in California. We estimated *F*_ST_ = 0.33 based upon haplotype frequencies. Our generalized linear binomial mixed model indicates a latitudinal cline in the distribution of haplotypes (*β* = −0.34, *p* < 0.0001) (Fig. 3A). The xeric haplotype is more likely to occur at sites in southern California, while northern locations show a higher frequency of the other two haplotypes.

## Discussion

Evidence from cpDNA sequences point to a minimum of three introductions from the Old World to California. We had originally expected at least two introductions because of the two previously described genotypes (Clegg & Allard, 1972), and indeed, separate introductions of the mesic and xeric allozyme genotypes are seen. However we also discovered a third haplotype that was mostly confined to Northern California. All three haplotypes in California are also observed in the western Mediterranean, indicating that there was a minimum of three distinct lineages introduced to California. The presence of multiple genotypes is a necessary precondition for recombination to contribute to adaptation/colonization success in the new range (Ellstrand & Schierenbeck, 2000).

The haplotypes within California show substantial large-scale among population structure largely due to a statewide shallow North-South cline (Fig. 3A). There are xeric haplotypes at Northern latitudes California, though their distribution is largely concentrated at southern latitudes. Mesic haplotypes are found mostly in the Northern region of California, but they are also found at many southern sites. This gradual cline of xeric haplotypes indicates that residual population structure remains from the 1970s, where during this time period xeric allozyme genotypes were predominant throughout California, and especially at southern latitudes (Clegg & Allard, 1972).

But there is also clear spatial mixing within populations. The presence of different haplotypes within populations increases the probability that hybrid recombination could occur through occasional outcrossing between different selfing lineages of *A. barbata*. At the within population level, spatial mixing of cpDNA lineages occurs most frequently in North-central populations close to San Francisco and the surrounding Bay Area. Interestingly, the Northern haplotype is quite distantly related to the other two Californian haplotypes (Fig. 2B), so it is possible that it is introducing additional new nuclear alleles to the Californian populations of *A. barbata*, (not present in the mesic and xeric genotypes) from which additional new recombinant genotypes may be emerging. The occurrence of all three genetic lineages within populations; (e.g., Geyserville, Bodega Bay, Marshall, and San Ardo) allows for the possibility that recombination could greatly enhance genetic variation.

The three introductions to California may have occurred at different times or concurrently. With too little divergence time having passed since the presumed original *A. barbata* introduction to California (∼200 years ago) (Jain & Marshall, 1967; Minnich, 2008), only small differences in our chosen cpDNA loci (i.e., no mutations have occurred in California, which would allow us to track movements within the invaded range), it is impossible to infer from the phylogeny which introduction came first. However, our previous field studies of the allozyme genotypes and their recombinants suggest that mesic genotypes have higher fitness than the xeric type (Latta, 2009). This leads to the prediction of the spread of genotypes derived from the mesic haplotype into areas formerly occupied by the xeric, especially in the northern region of California (Latta, 2009). This would suggest that the mesic was introduced after the xeric.

Since neither multi-locus allozyme combination occurred in the Old World (Garcia et al., 1989), the allozyme combinations described by Allard et al. (1972) were thought to be recombinants of alleles present in Spanish populations. These findings support the idea that recombination is relevant to colonization and that it contributes to adaptation. However, if these recombinant allozyme combinations were separately introduced to California, as we speculate above, then hybridization may have occurred in *A. barbata* populations colonizing habitats further south in South or Central America which were subsequently transported to California (Blumler, 2000). We cannot discern whether this initial recombination might have happened in California, or prior to arrival, because our geographic sampling is restricted to North America and the Mediterranean Basin. Further, as there were no new cpDNA haplotype mutants observed in California since leaving the Old World we would not be able to track the route of recombination or migration.

While flow cytometry data show that all Californian populations (accessions) are tetraploid, one conspicuous result was that the genome sizes (pg/2C values) of Californian tetraploid individuals were on average smaller (∼1.5%) than those of the Old World tetraploid individuals. It has been demonstrated that a reduced genome size for plants is adaptive in novel or stressful environments (as would be experienced during an invasion) and is associated with a number of phenotypic traits, such as rapid cell division during stem elongation, that facilitate invasion in *Phalaris arundinacea* (reed canarygrass) (Lavergne, Muenke & Molofsky, 2009). Some have argued that intraspecific variation may be an artifact of measurement error (Greilhuber, 1998; Doležel & Bartos, 2005). But our samples were grown under similar conditions and measured on the same machine, at the same time, with the same size standards. We therefore think that these highly statistically significant differences between Old World and Californian oats are genuine and worth further testing the hypothesis of reduced genome size in an invading population (Lavergne, Muenke & Molofsky, 2009).

### Old World

Our main purpose in examining the Old World was to determine the number and divergence of lineages introduced to California. While our Old World sampling was not intensive, it was more than sufficient to identify the main branches of the phylogeny (Nielsen & Slatkin, 2013). The mesic haplotype was widespread and the most commonly found haplotype in the Old World—occurring as far east as India and as far west as coastal Portugal. Based on our hypothesized rooting of our cpDNA tree, with the diploid *A. lusitanica* haplotypes, the mesic haplotype also appears to be one of the most derived. This is the opposite of what is predicted under coalescent theory, with the baseline expectation being that the most abundant and/or widespread haplotype is usually basal (Templeton, Routman & Phillips, 1995). This pattern suggests a recent and rapid spread of *A. barbata* individuals bearing the mesic cpDNA haplotype. Such a spread may have occurred if rapid expansion of the species range in the Old World had reduced genetic drift creating one widespread and abundant haplotype, as well as an excess of rare haplotypes (Excoffier, Foll & Petit, 2009), and our data appear to fit these expectations.

Although the large majority of our Old World accessions were tetraploid, a few were not. As every accession we obtained from germplasm repositories was originally identified and labeled as “*A. barbata*”, we used Peng et al.’s (2010) *trnL-F* phylogeny of *Avena* species to infer a plausible root (*Avena lusitanica*) consistent with the larger *Avena* phylogeny. The *Avena* samples we investigated in the Old World seem to be part of a polyploidy complex or a series of repeated polyploidizations with multiple origins, which is not uncommon for plants (Soltis & Soltis, 1999; Soltis et al., 2007; Husband, Baldwin & Suda, 2013). Near the hypothesized root of our tree, the Algerian samples from Djelfa and Batna are most closely related to the diploid *Avena damascena* sequences from Peng et al. (2010), yet our accessions are tetraploid, so we refer to these accessions as *Avena damascena-like* (Fig. 2B). We hypothesize that these tetraploids could potentially be an intermediate between the diploid *Avena damascena* (2x) and the derivative hexaploid *Avena fatua* (6x), based the placement of these species in Peng et al.’s (2010) phylogenies. Our phylogeny contains no accessions that matched the *trnL-F-trnF-R* sequence of *A. fatua*. The only hexaploids from our phylogeny were found in Iraq and Egypt, and are closely related to the tetraploid Northern *A. barbata* haplotype and they are distantly related to hexaploid *A. fatua* from Peng et al. (2010).

The Northern haplotype is tetraploid, but quite divergent in cpDNA sequence from the mesic and xeric haplotypes. One could argue that the Northern haplotype could be another tetraploid oat species such as *A. abyssinica* or *A. vaviloviana*, which cluster closely with *A. barbata* and have identical trnL-F sequences (Peng et al., 2010). However, it is difficult to separate the whole *A. barbata/abyssinica/vaviloviana* group and some authors have proposed it should all be considered *A. barbata* (Rajhathy & Thomas, 1974). These inferences, while worth revisiting to clarify phylogeographic hypotheses, are speculative, and were not the main focus of our study. Whether the Northern cpDNA haplotype is considered a separate species capable of hybridizing with A. barbata, or a distant lineage within one species complex does little to alter our main conclusion that there were three genetic lineages introduced to California. Within California there is almost certainly intermixing between the three introductions, especially in the North due to the presence of multiple haplotypes within populations. This sets up the possibility to test for recombination between the three different introductees, and the possible adaptive spread of a new recombinant genotype(s) to novel environments.

## Supplemental Information

10.7717/peerj.633/supp-1 Table S1 Tables of Genbank Accession numbers for accessions screened for chloroplast loci and chloroplast haplotype, and genome size information

10.7717/peerj.633/supp-2 Table S2 Table of Californian haplotypes screened with SSCP, and matching sequence information
